# Characteristics and surgical management of pure trapdoor fracture of the orbital floor in adults: a 15-year review

**DOI:** 10.1007/s10006-022-01099-2

**Published:** 2022-07-16

**Authors:** Ylenia Gugliotta, Fabio Roccia, Paolo Garzino Demo, Maria Beatrice Rossi

**Affiliations:** Division of Maxillofacial Surgery, Surgical Science Department, Città Della Salute E Delle Scienze Hospital, University of Turin, Corso A.M. Dogliotti 14, 10126 Turin, Italy

**Keywords:** Adult trapdoor fracture, Orbital trauma, Surgical timing, Epidemiology, Orbital floor fracture

## Abstract

**Purpose:**

This retrospective study aims to define the optimal timing of the surgical treatment of orbital floor trapdoor fractures (OFTFs) in adults according to clinical and radiological findings.

**Methods:**

From January 2006 to December 2020, 382 patients with isolated orbital floor fracture were admitted to the Division of Maxillofacial Surgery of Turin, Italy. The criteria for inclusion were age ≥ 16 years, preoperative computed tomography showing a linear (1a) or medial hinge fracture (1b), diplopia, and 6 months of follow-up data. Aetiology and mechanism of injury, presence of post-traumatic enophthalmos and oculocardiac reflex, time between trauma occurrence and surgery [stratified as < 24 h (urgent treatment), 24–96 h (early treatment), and > 96 h (late treatment)], days of hospitalisation, and clinical outcomes were examinated.

**Results:**

Twenty-four patients (18 males; mean age, 23.2 years) presented with OFTFs. The most common cause was sport injury (50%). Type 1a fracture was observed in eight patients (mean age, 19.5 years), type 1b fracture in 16 patients (mean age, 23.6 years). Urgent, early, and late treatments were performed in eight patients each. The mean time between trauma occurrence and surgery was 3,8 days (range: 0–17 days). Resolution of diplopia was observed 1 week after surgery in 10 patients, 1 month in 12. Diplopia persisted in 2 patient, both treated > 96 h after trauma.

**Conclusion:**

Although the number of patients was too small to define a standard protocol, the authors recommend early treatment of adult OFTFs to promote complete resolution of diplopia.

## Introduction

First described in 1965 by Soll and Poley [1], orbital floor trapdoor fractures (OFTFs) are commonly considered primary emergencies in paediatric maxillofacial traumatology, as well as a clinical variant described in 1998 by Jordan et al., defined “white eyed blowout” due to little clinical evidence of periorbital trauma [2].

Several authors have stated that in patients with vertical eye movement restricted caused by entrapment of the inferior rectus muscle or peri-muscular connective tissue, surgical treatment of OFTFs should be performed as soon as possible to reduce the risk of permanent diplopia [2–7]. However, there is no universal consensus regarding the indications and timing of surgical treatment for rare OFTFs in adults, as indicated by various case reports [8–16], and case series (typically involving few patients) [17–21].

Therefore, the purpose of this retrospective analysis was to define the indications and evaluate the optimal timing of the surgical treatment of OFTFs in adults, according to clinical findings and radiological fracture types. Accordingly, we analysed the long-term results of adult patients who underwent surgery for treatment of OFTFs in our hospital during the past 15 years.

## Methods

From January 2006 to December 2020, 2274 patients with maxillofacial fractures were admitted to the Division of Maxillofacial Surgery, Città della Salute e della Scienza Hospital, University of Turin (Turin, Italy); 382 of these patients exhibited isolated orbital floor fracture. The criteria for inclusion in the present retrospective study were as follows: age ≥ 16 years, availability of preoperative computed tomography scans with coronal view of the orbit, clinical evidence of diplopia and restricted eye movement, and at least 6 months of follow-up data.

**Fig. 1:**
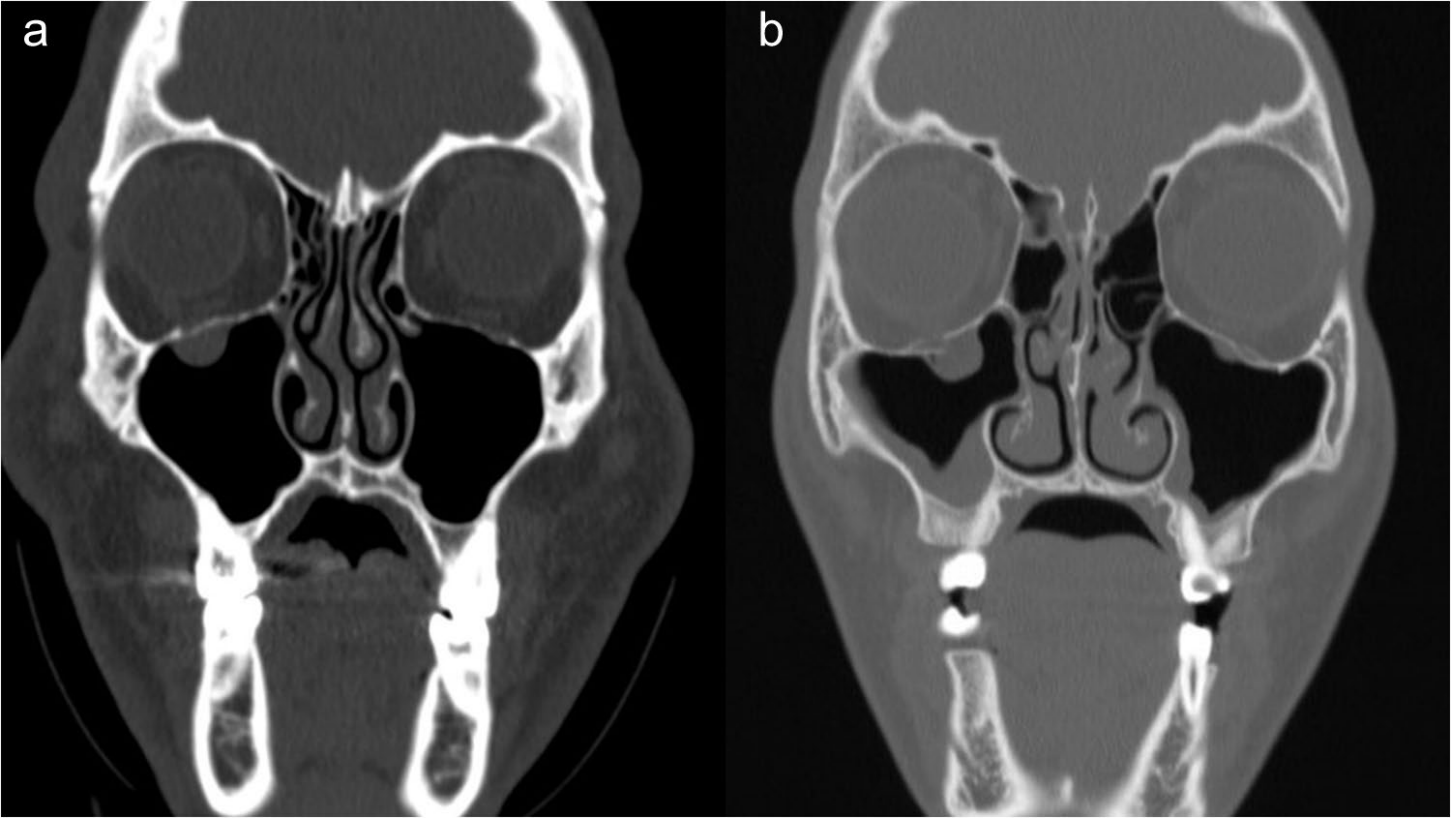
Preoperative CT scan, coronal view: **a** OFTF type 1a or linear fracture; **b** OFTF type 1b or hinged fractures according to Gerbino et al.

All the orbital floor fractures were radiologically classified according to Gerbino et al. [22] (Fig. 1) in type 1a, when a fracture line running along the infraorbital nerve canal to the retrobulbar area, with bone displacement smaller than the orbital floor thickness, and type 1b when a small medial part of the orbital floor was displaced downward further than the orbital floor thickness, along with a medial hinge.

Patients with incomplete clinical and radiological records, as well as those who had not completed postoperative follow-up examinations, were excluded from the study. The following data were examined in this study: trauma aetiology and mechanism, clinical evidence of post-traumatic enophthalmos, clinical signs of oculocardiac reflex, time between trauma occurrence and surgery [stratified as < 24 h (urgent treatment), 24–96 h (early treatment), and > 96 h (late treatment)] [2], days of hospitalisation, and clinical outcomes.

All surgical treatments were performed under general anaesthesia; administration of high-dose steroids was initiated before surgery and continued for 72 h postoperatively. Surgical repairs were performed using the subciliary or transconjunctival approach to expose the orbital floor. After each fracture had been visualised, the herniated tissue was gently dissected and reduced into the orbit. In all patients, Lyoplant (Braun, Tuttlingen, Germany) was placed on the orbital floor to cover the bone defect. At the end of each intervention, a duction test was performed. Patients were advised to return to the clinic for follow-up examinations, weekly for the first month after surgery and then monthly for 6 months. During follow-up visits, diplopia and ocular motility were recorded using the Hess–Lancaster screen.

## Results

Among 382 patients with pure orbital floor fractures, 24 (6.3%; 18 males and 6 females; mean age, 23.2 years (range: 16–47 years)] presented with OFTFs. OFTFs were caused by sports injuries in 12 patients, assault in 11 patients, and a fall in 1 patient (Table 1). The main mechanism of injury was a punch during a violent incident or collision with against another player during a sporting event (Table 2).

**Table 1:** Adult patients who underwent surgery for OFTF between 2006 and 2020

No	Age (yr)	Gender	Cause	Type of fracture	Interval to surgery (h)
1	16	M	Sport	1b	24–96
2	16	M	Violence	1a	< 24
3	17	M	Sport	1a	< 24
4	17	M	Violence	1b	< 24
5	17	M	Violence	1a	< 24
6	17	M	Sport	1b	24–96
7	18	M	Violence	1b	24–96
8	18	M	Fall	1b	24–96
9	18	F	Sport	1b	< 24
10	19	M	Sport	1a	> 96
11	20	M	Violence	1a	24–96
12	21	M	Violence	1b	< 24
13	21	M	Sport	1a	> 96
14	22	F	Violence	1b	> 96
15	22	M	Sport	1a	24–96
16	23	M	Sport	1b	24–96
17	24	M	Violence	1a	< 24
18	29	M	Violence	1b	< 24
19	29	M	Sport	1b	> 96
20	30	F	Sport	1b	24–96
21	30	F	Sport	1b	> 96
22	31	F	Violence	1b	> 96
23	34	F	Violence	1b	> 96
24	47	M	Sport	1b	> 96

*F* female, *M* male, *h* hour, *yr* years

**Table 2:** Type of OFTF in relation to the mechanism of maxillofacial trauma

		Type Ia	Type Ib	Total
Violence	Fist	4	6	10
	Kick	0	1	1
Sport	Other player	3	5	8
	Ground	1	2	3
	Object	0	1	1
Other	Ground	0	1	1

Type 1a fracture was observed in eight patients (mean age, 19.5 years), while Type 1b was observed in 16 patients (mean age, 23.6 years). Table 2 summarises the distribution of fracture types by injury mechanism. No patients older than 24 years exhibited type 1a OFTFs, and none exhibited post-traumatic enophthalmos or signs of oculocardiac reflex.

Surgery was performed as soon as possible, i.e. within 12 h of presentation; thus, the time between the occurrence of the trauma and surgery was influenced by the timing of presentation. Urgent, early, and late treatments were performed in eight patients each. The mean time between trauma occurrence and surgery was 3.8 days (range: 0–17 days), and the mean hospital stay was 3 days.

At the follow-up examination, resolution of diplopia was observed 1 week after surgery in 10 patients (5 had undergone surgery within 24 h of trauma, 1 within 24–96 h, and 4 > 96 h). Resolution of diplopia was observed at the 1-month follow-up examination in 12 of the remaining 14 patients (3 had undergone surgery within 24 h of trauma, 7 within 24–96 h, and 2 > 96 h). In the final two patients, diplopia persisted only in the upper field of gaze. Table 3 summarises the relationships among fracture type, timing, and outcomes.

**Table 3:** Relationship among time of surgery, type of OFTF, and outcome (resolution of diplopia)

Time of surgery (h)	Type of fracture	No. of patients	1 week	1 month	Not solved after 6 months
< 24	1a	4	2	2	0
	1b	4	3	1	0
24–96	1a	2	1	1	0
	1b	6	0	6	0
≥ 96	1a	2	2	0	0
	1b	6	2	2	2
Total		24	10	12	2

## Discussion

Progressive loss of elasticity of bone tissues in adults, particularly involving the orbital floor, causes the bone to become more susceptible to displaced or comminuted fractures, and is often associated with other facial fractures [2, 7, 18, 20]. Therefore, pure fractures (i.e. with minimal displacement of the orbital floor or entrapment of the orbital soft tissues) are rare among adults, especially compared with the paediatric population [4, 7, 19, 21], in which the orbital wall is likely to bend, crack, and then to return to its normal position causing the orbital content to be trapped into the fracture [2, 9].

In this retrospective analysis of all pure orbital floor fractures surgically treated in our hospital, OFTFs were present in 6.3% of patients; this was similar to the rate reported by Takahashi et al. [7]. This rare type of fracture mainly affects young adult males and boys in the second decade of life, according to published case reports [8–16], and case series [17–21], involving patients over 30 years of age (Table 4). The most frequent aetiologies of this type of orbital fracture in our study were similar to the literature (i.e., assault and sports injuries). In addition, consistent with numerous reports [11, 13, 17, 18], the trauma aetiology generally did not involve road traffic accidents [15, 18, 20], or falls [8], which represent the main causes of maxillofacial fractures. In this study, direct trauma to the orbital region results from a medium- or low-speed impact (generally following an assault or collision with an opponent during a sporting event), which produce twofold more type 1b than type 1a fractures. Furthermore, type 1b fractures increase in frequency with age, such that no 1a fractures have been reported in patients older than 24 years of age [21]. Notably, oculocardiac reflex and enophthalmos were not encountered in our sample population; these manifestations are very rare in adults.

**Table 4:** Summary of adult OFTF published in the recent literature

	Gender	Age	Cause	Presentation	Time of surgery (days)	Outcome
Sires et al. (1998)	M	20	Fall	Diplopia, OR	3	Diplopia
Kakizaki et al. (2005)	M	20	Blunt trauma (elbow)	Diplopia, OR	16	Diplopia sup
Kum et al. (2009)	M	37	Work	Diplopia	21	Diplopia sup
Mehanna et al. (2009)	M	17	Assault	Diplopia, OR	24	No diplopia
	M	21	Assault	Diplopia, OR	6	Diplopia
Yano et al. (2010)	M	16	Assault	Diplopia	5	No diplopia
	M	17	Sport	Diplopia	12	ND
	M	18	Sport	Diplopia	8	ND
	M	24	Sport	Diplopia	10	ND
Ethunandan and Evans. (2011)	M	16	RTA	Diplopia, OR	3	Diplopia sup
	F	16	Sport	Diplopia, OR	7	No diplopia
	F	17	Assault	Diplopia	11	Diplopia sup
	M	21	Assault	Diplopia, OR	< 1	No diplopia
	M	29	Sport	Diplopia	17	Diplopia sup
	F	53	Assault	Diplopia, OR	41	No diplopia
Summers et al. (2017)	M	26	Assault	Diplopia	ND	ND
Papadiochos et al. (2019)	M	29	Assault	Diplopia, OR	5	No diplopia
Said et al. (2019)	M	23	ND	Diplopia	ND	ND
Karthik et al. (2019)	M	16	Assault	Diplopia	11	Diplopia sup
	M	17	Assault	Diplopia	> 1	No diplopia
	M	24	Assault	Diplopia	6	Diplopia sup
	F	27	RTA	Diplopia	48	No diplopia
	M	29	RTA	Diplopia	< 1	No diplopia
Brasileiro et al. (2020)	F	26	RTA	Diplopia, OR	1	No diplopia
Gowda et al. (2020)	F	25	ND	Diplopia	3	Diplopia sup
Al-Qattan et al. (2021)	M	25–50	ND	Diplopia	< 2	No diplopia

*F* female, *M* male, *ND* not defined, *OR* oculocardiac reflex, *RTA* road traffic accident

Although Scolozzi et al. [23] reported that guidelines in several centres continue to include a waiting period of 14 days before surgery in patients with blowout fractures who exhibit persistent diplopia in primary gaze with restricted ocular motility, there remains a general consensus supporting urgent surgical treatment of OFTFs in both adult and paediatric patients [2–7]. In a series of 10 adult trapdoor fractures (including 7 pure), all surgically treated within 48 h, Al-Qattan and Al-Qattan did not encounter postoperative diplopia [21]. Similarly, no cases of diplopia were reported by Mehanna et al. [11] or Brasileiro et al. [15], among patients who had undergone surgery within 24 h. Karthik et al. [20] observed that among four adult cases of OFTF, the two patients who experienced the greatest delays between trauma and surgery (8 and 9 days, respectively) exhibited the worst outcomes; Ethunandan et al. [18]reported similar outcomes in 2 patients surgically treated at 11 and 17 days after trauma. Takahashi et al. [7] suggested that a long interval between trauma and surgery could promote fibrotic and necrotic changes in entrapped tissues, causing greater postoperative restriction of ocular mobility. In contrast, Kwon et al. [4] suggested that OFTFs with severe muscle limitations should be surgically treated in adult patients within 5 days after trauma; in a recent review of the literature, Papadiochos et al. [13] observed that non-emergent OFTFs in adults showed satisfactory outcomes even when treatment was delayed beyond the 48 or 72 h recommended for paediatric patients. Various studies [7, 13, 17, 24, 25] have suggested that the difference in surgical timing between adults and children could be explained by the better prognosis for type 1b OFTFs, which are more common in adults; the larger amount of trapped tissue in children (i.e. inferior rectus muscle within the fracture gap), rather than the considerable differences in necrosis and fibrosis, makes treatment more urgent in these patients.

Although the low number of OFTFs in this study may have obscured statistically significant differences in the prognoses of patients with type 1a versus 1b fractures, analysis of treatment timing showed that only two patients (both surgically treated > 96 h after trauma) did not exhibit diplopia resolution at the 6-month follow-up examination. In our hospital, the treatment timing for paediatric and adult patients with OFTFs is similar; thus, patients aged over 15 years undergo surgery as soon as possible. This early surgery can easily (and often non-traumatically) release the muscle and/or entrapped orbital tissues.

## Conclusion

This retrospective study showed that pure OFTFs in adults almost exclusively affected young boys and males under 30 years of age; moreover, they were caused by low-to-medium energy impacts (almost exclusively sports injuries or violent assaults) and type 1b fractures were more common with advancing age. Finally, although the number of patients was too small to define a standard treatment protocol, the authors recommend early treatment of this rare type of orbital fracture, because urgent and early treatments can achieve complete resolution of diplopia.
